# Supplementary material for: Analysis of cardiomyocyte clonal expansion during mouse heart development and injury
Source: Nat Commun. 2018 Feb 21;9:754. doi: 10.1038/s41467-018-02891-z (PMC5821855; doi:10.1038/s41467-018-02891-z)
Supplement: Supplementary file 1 — Supplementary Information [file 41467_2018_2891_MOESM1_ESM.pdf]

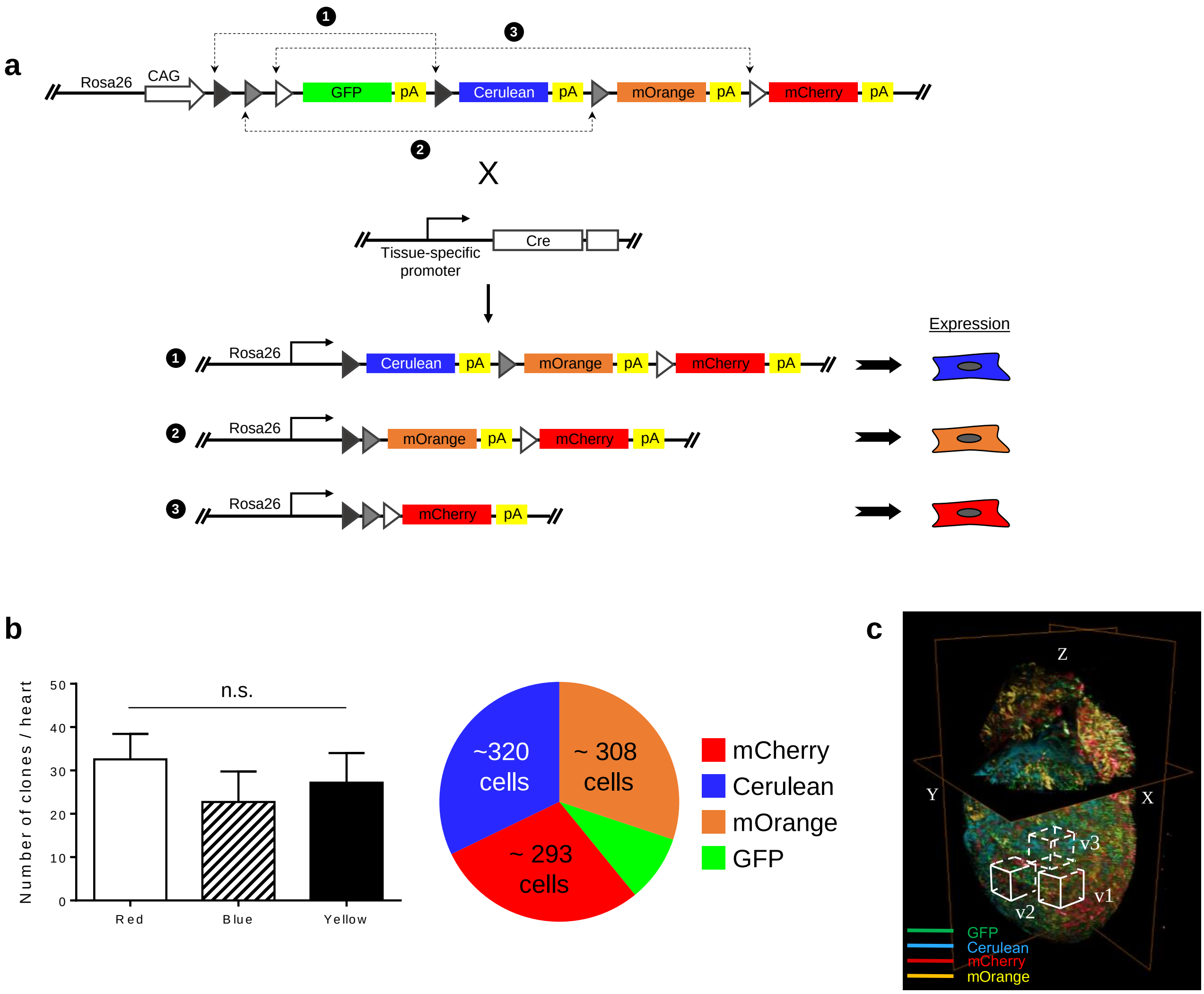

Supplementary Figure 1: The Rainbow model provides a precise, direct method to study clonal expansion of cardiac progenitors and CMs within the developing heart. a, Schematic representation of the Rainbow reporter system. Expression of Cre induces random recombination between mutated paired lox P sites (black, grey and white triangles) leading to expression of Cerulean, mOrange and mCherry respectively. **b-c,** Quantification of the number of labeled clones per heart from 2D sections of P1 hearts (n=11) **(b)** and 3D imaging of a αMHC^{Cre} ; $R26^{VT2/GK}$ P1 heart **(c)** demonstrates equal representation of Cerulean, mOrange and mCherry. All measurements shown are depicted as mean ± s.em.

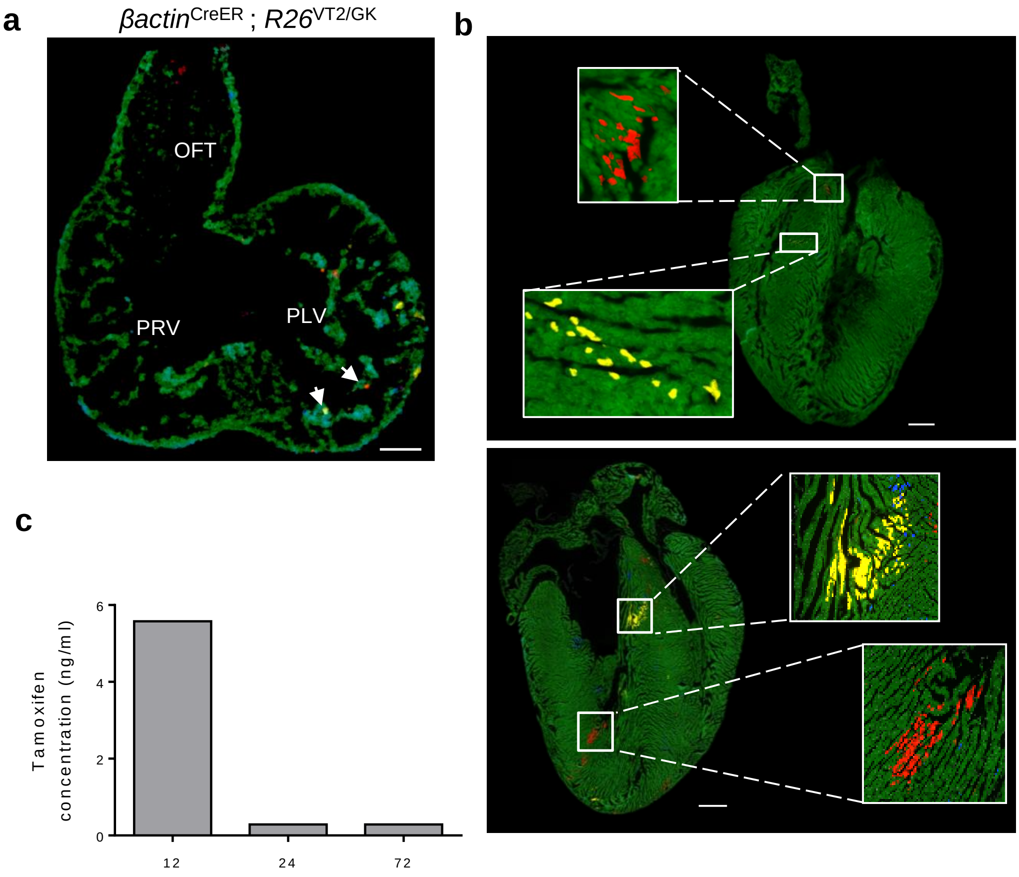

Supplementary Figure 2: Single low dose of tamoxifen induces rare recombination events. a, Representative fluorescent microscope image of *βactin*^{CreER} ; *R26*$^{VT2/GK}$ E10.5 hearts 24hrs post tamoxifen administration. Arrows indicate single recombination events. Scale bar 100 μm.**b,** Representative images of adult hearts with sparse clones. Recombination was induced at E9.5. Insets show magnification of boxed areas. Scale bar 500μm. **c**, Tamoxifen is removed from the circulation within 24 hours after treatment. Blood serum levels of tamoxifen measured 12, 24, and 72 hours following administration. Tam, tamoxifen; OFT, outflow tract; PRV, primitive right ventricle; PLV, primitive left ventricle.

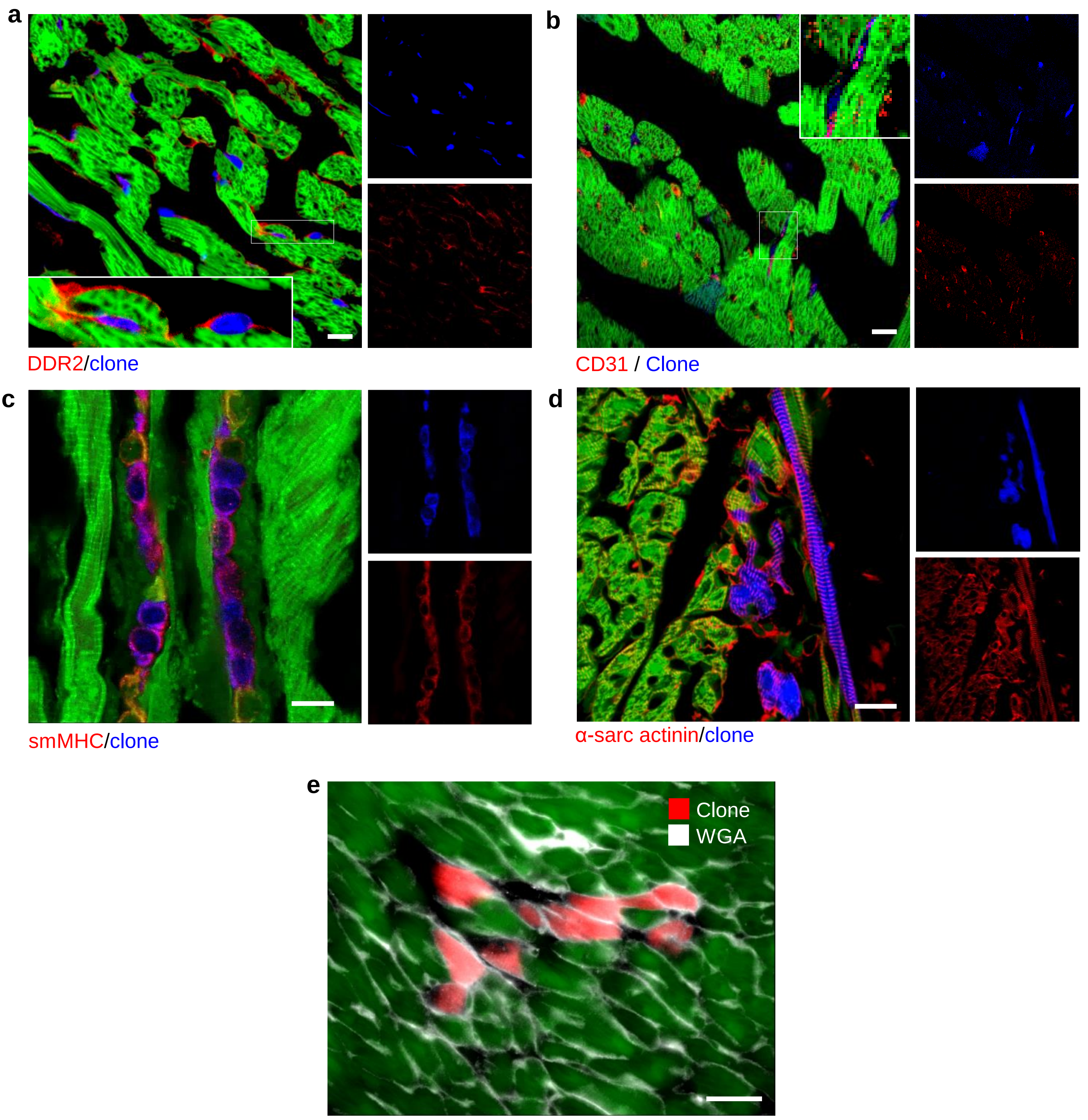

Supplementary Figure 3: Immunohistochemical staining of clones. . Immunohistochemical staining of *βactin*^{CreER}; *R26*$^{VT2/GK}$ clones positive for **a,** the fibroblast marker, DDR2, **b,** endothelial cell marker, CD31, **c,** smooth muscle myosin heavy chain, smMHC (scale bar 50 μm), and **d,** CM, α-sarcomeric actinin. Insets show close ups of the co-localization. **e,** WGA (white) was used to delineate individual cells within a clone (red pseudocolor) for counting purposes. Scale bar 100 μm unless otherwise denoted.

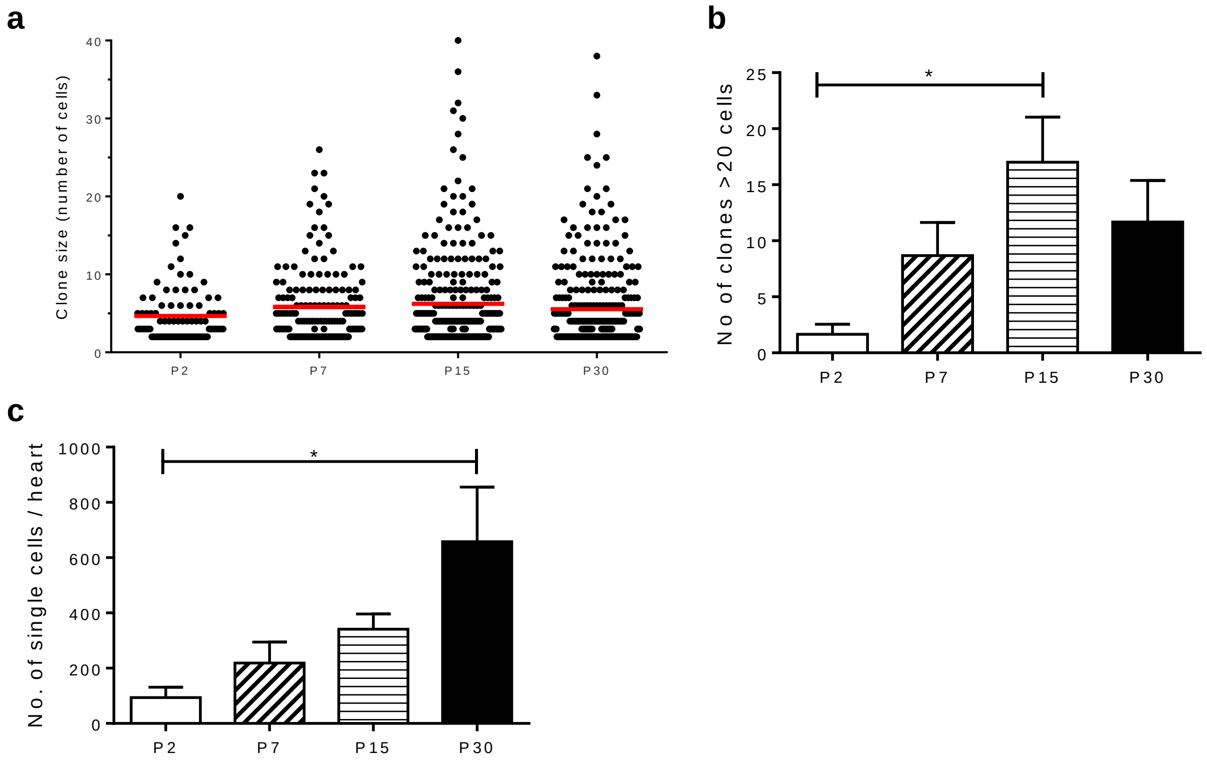

Supplementary Figure 4: Clonal expansion occurs mainly during embryonic development. a, Dot plot analysis of clonal sizes in *βactin*^{CreER} ; *R26*$^{VT2/GK}$ hearts labeled at E9.5 and analyzed at P2, P7, P15, and P30 (n=3). **b**, Number of clones > 20 cells progressively increase up to P15 and declines by P30. **c**, Number of single cells increases over time (n=3). All measurements shown are depicted as mean ± s.em.

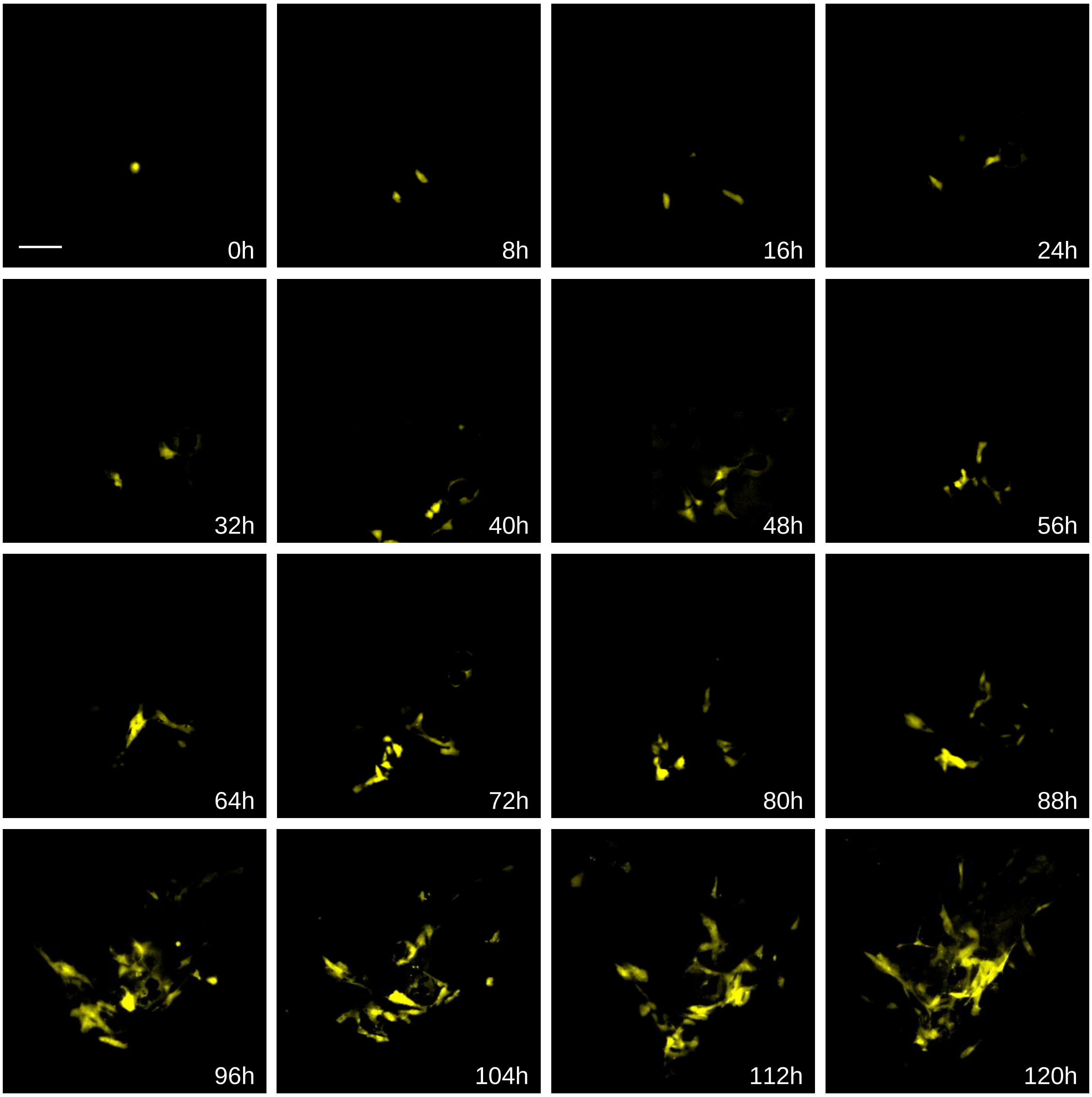

Supplementary Figure 5: "Rainbow-labeled" cardiac cells expand clonally *in vitro*. Time-lapse imaging of a single rainbow-labeled cell (yellow pseudocolor) forming a clone *in vitro*. Monitoring was performed 0-120 hours after plating. Scale bar 100μm.

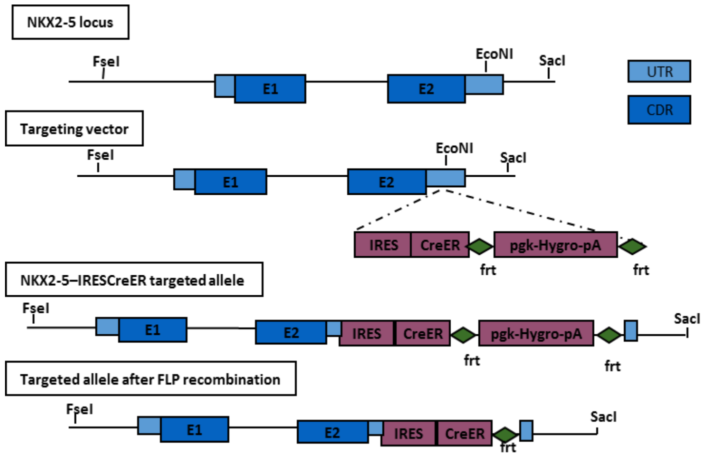

Supplementary Figure 6: Generation of the Nkx2.5-CreER construct. Maps of the Nkx2.5 wild-type allele, gene targeting construct and resultant mutant alleles before (Nkx2.5IRES-CreER-pgkHYGRO) and after (Nkx2.5IRES-CreER) flp-mediated deletion. Several stable clones were generated and founders of this new strain (Nkx2.5-CreER) were backcrossed onto C57BL/6 mice.

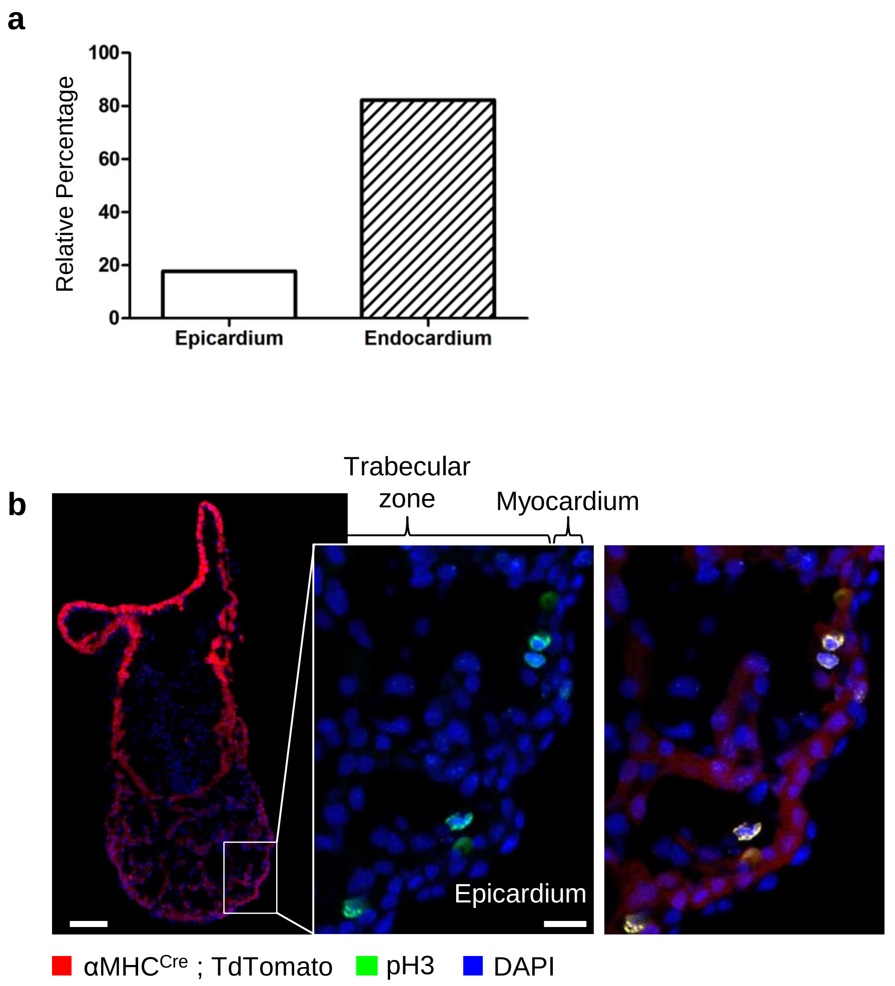

Supplementary Figure 7: CM clones are mainly localized near the endocardium. a, Quantification of CM clones based on their proximity to either the epicardium or the endocardium. Tamoxifen was administered at E9.5 and hearts were analyzed at P2. **b,** Immunohistochemical staining for phospho-Histone H3 (pH3 in green) of a E9.5 *αMHC*^{Cre} ; TdTomato heart. Scale bar 100μm. Inset shows magnification of boxed area, scale bar 20μm.

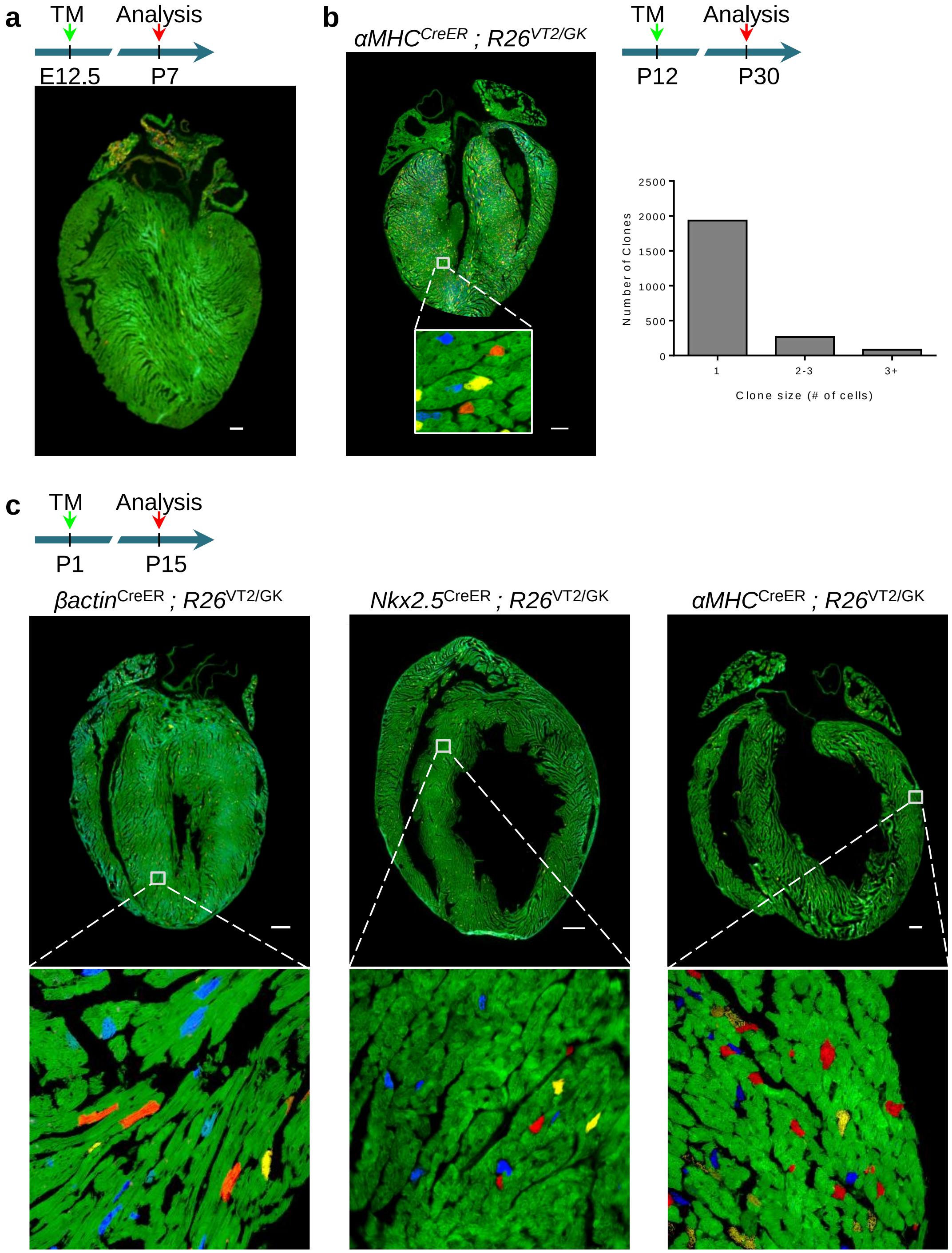

Supplementary Figure 8: Postnatal CMs exhibit limited proliferative capacity in the absence of injury. a, *αMHC*^{CreER} ; *R26*$^{VT2/GK}$ mouse heart labeled at E12.5 and analyzed P7 showing the distribution of αMHC primarily in the atria and to a lesser extent in the ventricles, scale bar = 200 μm. **(b, left)** Representative image of *αMHC*^{CreER} ; *R26*$^{VT2/GK}$ P30 heart labeled at P12 showing the dominance of single Rainbow-labeled cells, scale bar 500 μm. **(b, right)** Quantitation of clone size and number within *αMHC*^{CreER} ; *R26*$^{VT2/GK}$ mouse hearts labeled at P12 and analyzed P30 (n=4, 8 images at 40X magnification analyzed per section, 5 sections/heart).**c**, Representative confocal microscope images of *βactin*^{CreER} ; *R26*$^{VT2/GK}$, *Nkx2.5*^{CreER} ; *R26*$^{VT2/GK}$, and *αMHC*^{CreER} ; *R26*$^{VT2/GK}$ P15 hearts labeled at P1. Insets show up close view of boxed areas. Scale bar 500 μm.

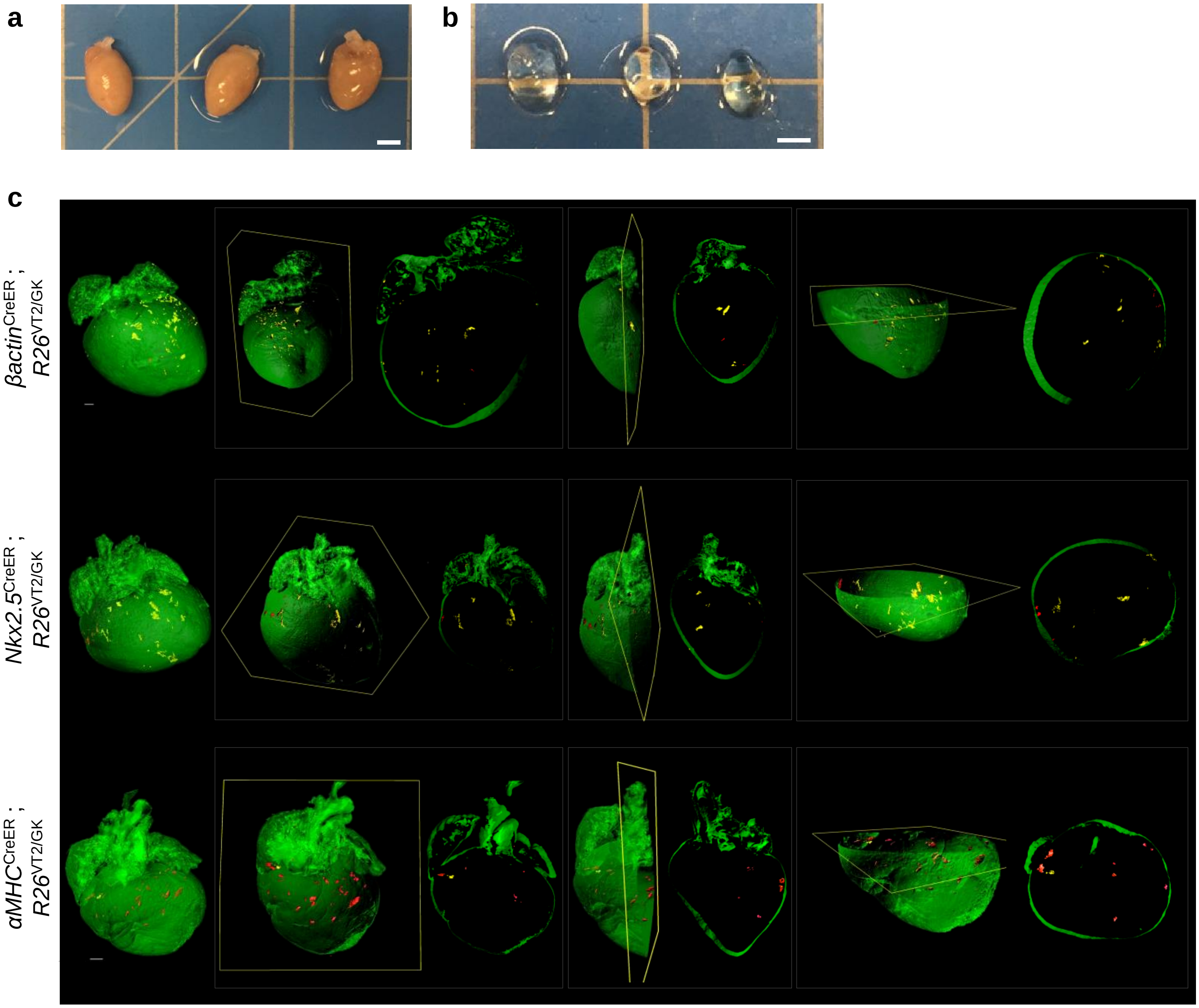

Supplementary Figure 9: Three dimensional imaging of cleared whole neonatal mouse hearts using the CLARITY method. P2 hearts prior to CLARITY **(a)** and post CLARITY **(b)**, scale bars = 2 mm. **c,** 3 dimensional rendering of cleared P2 hearts from *βactin*^{CreER} ; R26$^{VT2/GK}$, *Nkx2.5*^{CreER} ; R26$^{VT2/GK}$, and *αMHC*^{CreER} ; R26$^{VT2/GK}$. Examples of sagittal, coronal, and axial planes captured using Imaris 7.7.2 software (Bitplane AG, Zurich).

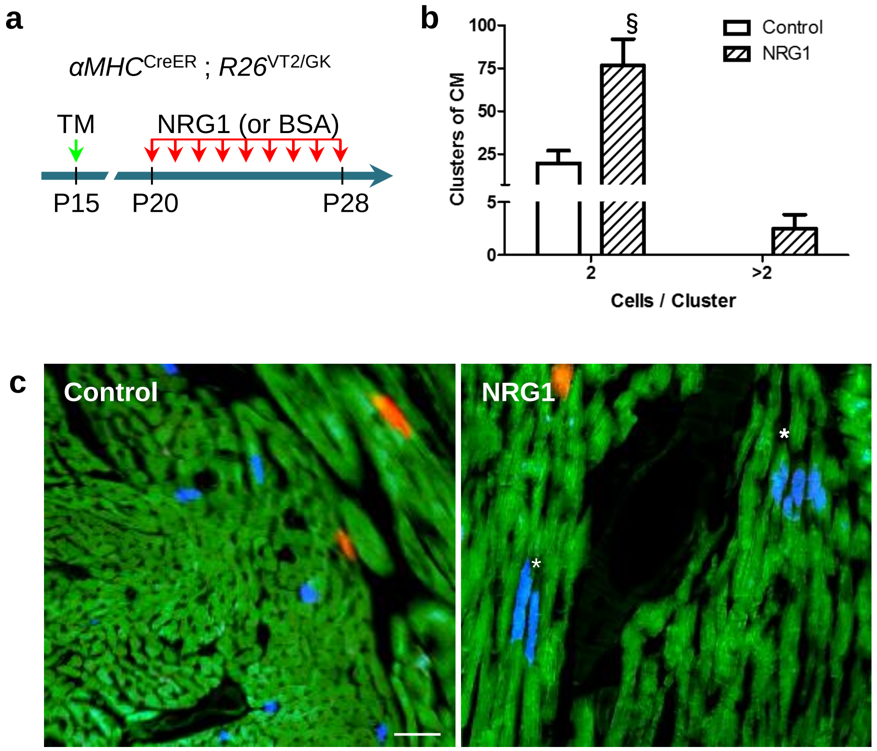

Supplementary Figure 10: NRG1 treatment induces postnatal CM proliferation. a, Experimental design of NRG1 treatment. Arrow indicates tamoxifen administration at P15. NRG1 (n=4) or BSA control (n=3) were given daily to *αMHC*^{CreER} ; *R26*$^{VT2/GK}$ mice from P20 to P28. **b,** Quantification reveals increased formation of CM clusters following NRG1 treatment. **c,** Representative fluorescent microscope images of control and NRG1-treated hearts. Stars indicate clusters of CMs. § NRG1 vs control, $p<0.05$. Scale bar 50 μm. All measurements shown are depicted as mean ± s.em.

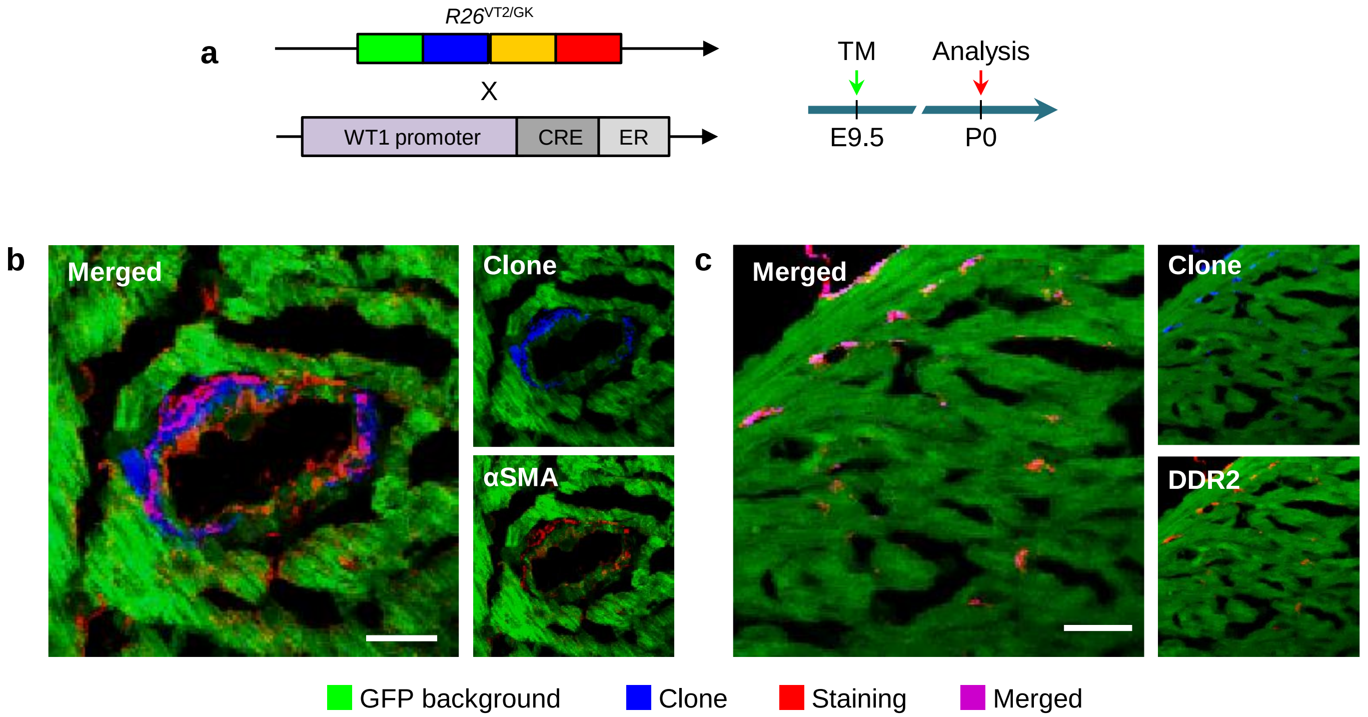

Supplementary Figure 11: Smooth muscle cell and fibroblast clones of WT1 origin. a, Experimental design. *Wt1*^{CreER} ; *R26*$^{VT2/GK}$ mice received tamoxifen at E9.5 and were analyzed at P0. **b-c,** Representative confocal microscope images of **b**, a clone (blue) consisting of smooth muscle cells (α-SMA in red) and **c**, a clone of fibroblasts (DDR2 in red). Insets show the individual pseudocolors in GFP background. Scale bars 20 µm.

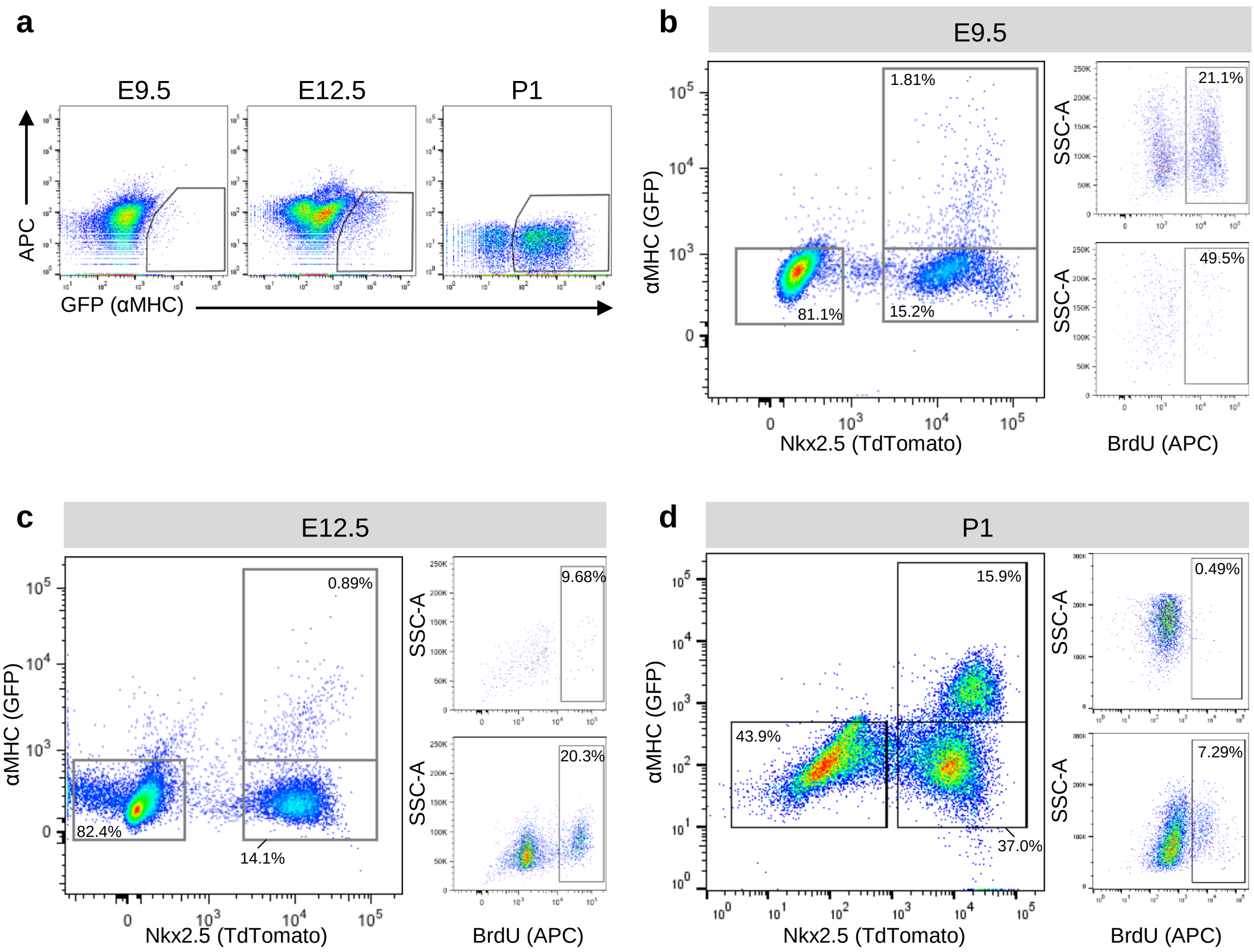

Supplementary Figure 12: BrdU incorporation in CMs during development. a, Representative flow cytometric analysis of αMHC-GFP cells sorted for qPCR or single cell analysis. Gated areas show cells selected for sorting. **b-d,** Representative flow cytometric analysis plots of BrdU incorporation in *Nkx2.5*$^{+/Cre}$*; R26R-Tdt*$^{+/fl}$*; αMHC*$^{+/GFP}$ hearts at **(b)** E9.5, **(c)** E12.5, and **(d)** P1.

a b

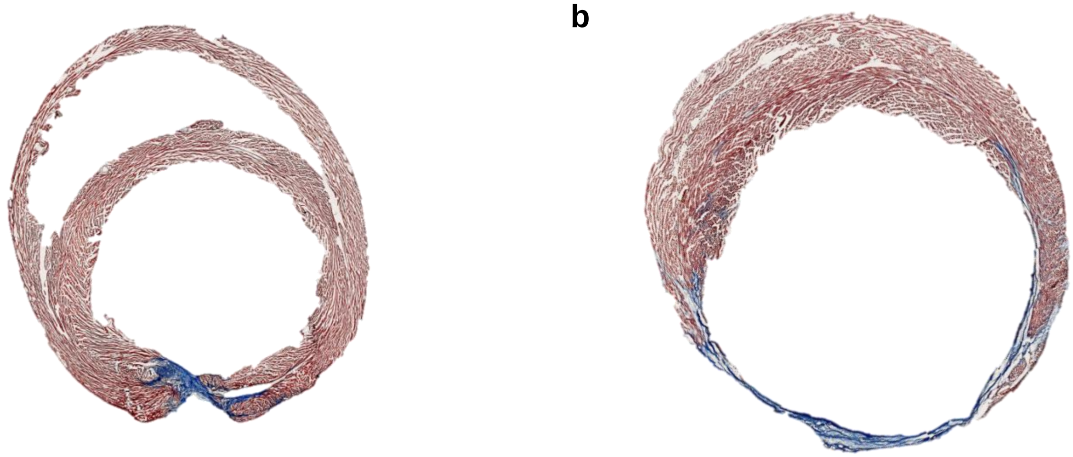

Supplementary Figure 13: Mason's trichrome staining of infarcted hearts. Representative images of a heart section 21 days following LAD ligation at **(a)** P1 and **(b)** 2 months of age. A small area of fibrosis (blue) is still apparent **(a)** after neonatal injury while injury in adult results in large scar formation **(b)**.

a

	E9.5	E12.5	P1
Cluster 1	23	1	0
Cluster 2	13	7	0
Cluster 3	6	27	1
Cluster 4	1	1	42
**Total**	**43**	**36**	**43**

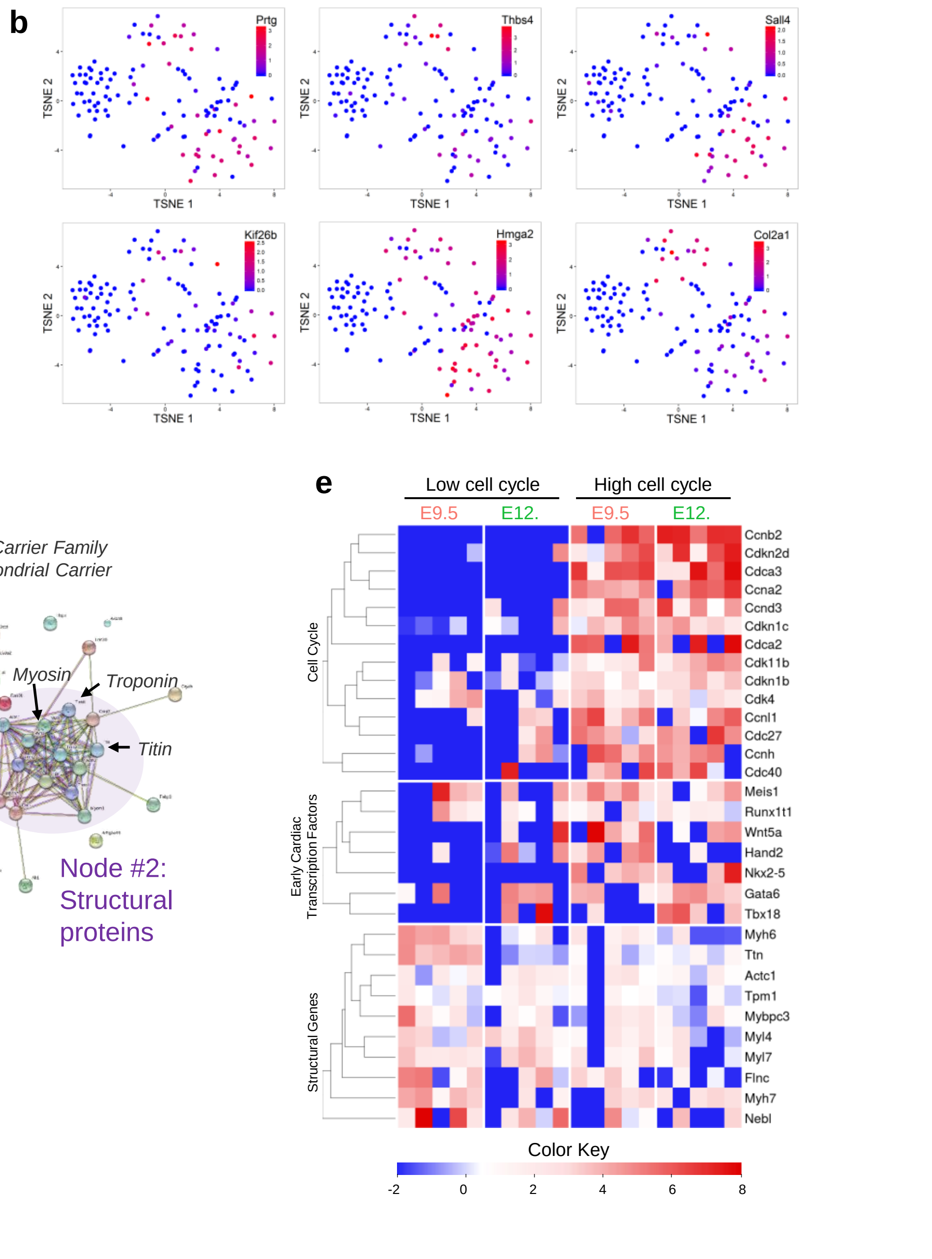

Supplementary Figure 14: Transcriptional profiling of CMs during early embryonic and postnatal development. a, Table depicting numbers of single cells found within each km cluster. **b,** Expression of genes found only in E9.5 cells (*Thbs4, Kif26b, Col2a1, Prtg)* or were highly enriched (*Sall4, Hmga2)* within cells from this timepoint. **c**, Venn diagram depicting the number of differentially expressed genes between P1 cluster and P1 outlier. **d**, Search Tool for the Retrieval of Interacting Genes/Proteins (STRING) analysis comparing the top 50 genes more highly expressed in the P1 cluster compared to P1 outlier. Two nodes were identified encoding for genes of metabolism (bronze circle) and structural proteins (purple circle). **e,** Single cells at E9.5 and E12.5 were selected based on low or high cell cycle activity, as determined by expression levels of common cell cycle genes. Genes encoding for cardiac structural proteins and transcription factors were examined within the selected cells showing a possible link between cell cycle activity and CM maturity. **f,** RNA Sequencing Analysis Workflow. RNASeq data was mapped with OLego version 1.1.5 and normalized using Transcripts per Millions analysis. featureCounts version v1.5.0-p2 was used to estimate total number of reads. Expression levels were then log transformed using algorithm depicted. ** $p < 0.01$. Mean ± SEM.

Supplementary Table 1: List of genes for used for heatmap in Figure 5b.

Gene Group	List of Genes			
Muscle / Cardiac Cell Differentiation	Myh9	Tiparp	Lgals1	Mbnl1
	Myl6	Myh6	Tbx18	Ttn
	Myh10	Actg1	Dlg1	Dicer1
	Tgfb2	Foxp1	Sort1	Ppp3ca
	Tbx5	Tagln2	Rhoa	Tmod1
Gene Silencing / Histone Modification	Tyms	Hells	Coq5	Rrp8
	Hdac1	Sirt6	Trmt112	Brcc3
	Ilf3	Gspt1	Ncbp2	Mat2a
	Smarca5	Gamt	Kdm6b	N6amt1
	Ehmt1	Dhfr	Wbscr22	Tnrc6b
	Prmt7	Ezh2	Tgs1	
Cell Cycle	Cks1b	Ccnd1	Smad3	Anapc11
	Myh9.1	Haus7	Nde1	Rbbp4
	Cdk4	Tgfb2.1	Fam33a	Rcc2
	Trp53	Spin1	Dhfr.1	Rbm7
	Cdca8	Pmf1	Calm2	Bcl2
	Ranbp1	Tipin	Erh	E2f3
	Mcm7	Hells.1	Cdk11b	Haus1
	Myh10.1	Cenpe	Ak1	Msh2
	Gmnn	Tacc3	Actb	Nedd1
	Sik1	Myh6.1	Mfn2	Gak
	Cfl1	Actg1.1	Ccar1	Rac1
	Mcm2	Anapc5	Ccnh	Rhoa.1
	Nudc	Ccnd3	Npm1	Mapre1
	Mcm5	Haus8	4933424B01Rik	Lrrcc1
	Tpx2	Cdk7	Rpl24	
	Hcfc1	Cdk2	Tcf3	
Metabolism	Gbas	Uqcrq	Cycs	Atp5j
	Uqcrfs1	Atp5h	Atp5l	Cox7c
	Uqcrb	2410091C18Rik	Atp5o	Cox6c
	Myh7	Itpr1	Cox6b1	
Cell Growth / Migration	Cdk4.1	Tgfb2.2	Hif1a	Bcl2.1
	Trp53.1	Tbx5.1	Dlg1.1	Rac1.1
	Gtpbp4	Celf1	Mkks	
Ion Channels / Transporters	Impdh2	Pkm2	Atp1b2	Mat2a.1
	Tnfaip1	Cdk2.1	Abcc9	Gmpr2
Muscle / Cardiac Cell Structure & Contraction	Ankrd1	Tbpl1	Hmgn2	Ank2
	Myh9.2	Polr1e	Itpr1.1	Ldb3
	Actn1	Haus7.1	Abcc9.1	Ttn.1
	Tpm2	Zdbf2	Cycs.1	Hist1h2an
	Myl6.1	Myh7.1	Gnpat	Dicer1.1
	Cnn3	Cenpe.1	Rpl24.1	Rpl9
	Myh10.2	Myh6.2	Dlg1.2	Cacna1c
	Cald1	Actg1.2	Hist1h2ab	Ppp3ca.1
