# Supplementary material for: Analysis of cardiomyocyte clonal expansion during mouse heart development and injury
Source: Nat Commun. 2018 Feb 21;9:754. doi: 10.1038/s41467-018-02891-z (PMC5821855; doi:10.1038/s41467-018-02891-z)
Supplement: Supplementary file 2 — Description of Additional Supplementary Files [file 41467_2018_2891_MOESM2_ESM.docx]

**Description of Additional Supplementary Files**

File Name: Supplementary Movie 1

Description: Serial frozen sections of an E15.5 β-actin^CreER^; *R26*^VT2/GK^ heart reconstructed to illustrate cardiomyocyte clones.

File Name: Supplementary Movie 2

Description: Red and yellow clones rendered from an *Nkx2.5*^CreER^; *R26*^VT2/GK^ mouse heart labeled at E12.5 using the CLARITY technique.
